# Suppression of mucosal Th17 memory responses by acellular pertussis vaccines enhances nasal *Bordetella pertussis* carriage

**DOI:** 10.1038/s41541-020-00270-8

**Published:** 2021-01-08

**Authors:** Violaine Dubois, Jonathan Chatagnon, Anaïs Thiriard, Hélène Bauderlique-Le Roy, Anne-Sophie Debrie, Loïc Coutte, Camille Locht

**Affiliations:** 1 CNRS, Inserm, CHU Lille, Institut Pasteur de Lille, U1019—UMR9017—CIIL—Center for Infection and Immunity of Lille, Univ. Lille, 59000 Lille, France; 2 CNRS, Inserm, CHU Lille, Institut Pasteur de Lille, US41—UMS 2014—PLBS, Univ. Lille, 59000 Lille, France

**Keywords:** Vaccines, Cellular immunity

## Abstract

Pertussis has made a spectacular rebound in countries that have switched from whole-cell (wPV) to acellular pertussis vaccines (aPV). Here, we show that, unlike wPV, aPV, while protective against lung colonization by *Bordetella pertussis* (Bp), did not protect BALB/c mice from nasal colonization, but instead substantially prolonged nasal carriage. aPV prevented the natural induction of nasal interleukin-17 (IL-17)-producing and interferon-γ (IFN-γ)-producing CD103^+^ CD44^+^ CD69^+^ CD4^+^-resident memory T (T_RM_) cells. IL-17-deficient, but not IFN-γ-deficient, mice failed to clear nasal Bp, indicating a key role of IL-17^+^ T_RM_ cells in the control of nasal infection. These cells appeared essential for neutrophil recruitment, crucial for clearance of Bp tightly bound to the nasal epithelium. Transfer of IL-17^+^ T_RM_ cells from Bp-infected mice to IL-17-deficient mice resulted in neutrophil recruitment and protection against nasal colonization. Thus, aPV may have augmented the Bp reservoir by inhibiting natural T_RM_ cell induction and neutrophil recruitment, thereby contributing to the pertussis resurgence.

## Introduction

Pertussis, also known as whooping cough, is a highly contagious respiratory disease, mainly caused by *Bordetella pertussis* (Bp). The most severe manifestations of pertussis occur in young children, but pertussis also affects adolescents, adults, and the elderly^1^. After the implementation of whole-cell pertussis vaccines (wPVs) combined with diphtheria- and tetanus-toxoid-based vaccines, referred to as DTP vaccines, in the 1950s and 1960s, the disease burden markedly lessened. However, concerns about the safety of wPV led to the replacement of DTP vaccines with diphtheria tetanus acellular pertussis vaccines (aPVs) in most industrialized countries in the 2000s. Less reactogenic than wPV, aPVs contain one to five purified Bp antigens and are highly effective in preventing pertussis disease^2^.

Despite high vaccination coverage, pertussis is now re-emerging in several areas of the world^3,4^. Possible reasons that may contribute to this resurgence include improved diagnosis, the emergence of antigenic variants that escape vaccine-mediated immunity, suboptimal and fast waning immunity, especially after aPV vaccination^5–7^, and the inability of the current vaccines to prevent infection and transmission of Bp^8,9^.

Improving our understanding of pertussis pathogenesis and how vaccines may modulate Bp interactions with its host is needed to enhance whooping cough control. Although mice do not develop the characteristic cough associated with the disease, they share many features of human whooping cough^10,11^ and have been used in many studies to decipher immune mechanisms and various aspects of Bp infection^12^.

Bp clearance from the lungs of immunized mice has been shown to correlate with pertussis vaccine efficacy in children^13–16^. Similar to infection, immunization with wPV was associated with the induction of strong T helper type 1 (Th1)/Th17 immune responses, whereas aPV preferentially induces Th2 cells in mice^13,14,16,17^. Similar observations were reported in humans and in non-human primates^8,18^. These Th subsets are mainly identified by signature cytokines such as interferon-γ (IFN-γ) (Th1), interleukin-17 (IL-17) (Th17), and IL-4, IL-5, and IL-13 (Th2). Complementary roles for cellular and humoral immunity in protection have been identified in the mouse model, showing that both antibodies and T cell responses are important for protection. In prototypical Th1 C57BL/6 mice, lung infection by Bp is prevented by the accumulation of tissue-resident memory T (T_RM_) cells in the lungs^19,20^, and induction of T_RM_ cells in the nose negatively correlates with nasal bacterial burden^20^. While vaccination with aPV strongly reduces the Bp load in the lungs, it does not protect against nasal carriage of Bp in the nasal cavity of mice^21^, consistent with its inability to do so in a non-human primate model^8^.

In this study, we took advantage of Th2-prone BALB/c mice to model the impact of aPV immunization on immune responses to Bp infection. This model was chosen as it reflects the situation in newborns, who are biased toward a Th2-type differentiation^22^. We report here that aPV immunization, while providing strong protection in the lungs, not only fails to protect against nasal colonization by Bp but also prevents natural clearing of Bp infection in the noses of BALB/c mice by suppressing Th17 T_RM_ cell responses and ensuing neutrophil recruitment.

## Results

### aPV immunization prolongs nasal carriage of Bp in Th2-prone mice

**Fig. 1:**
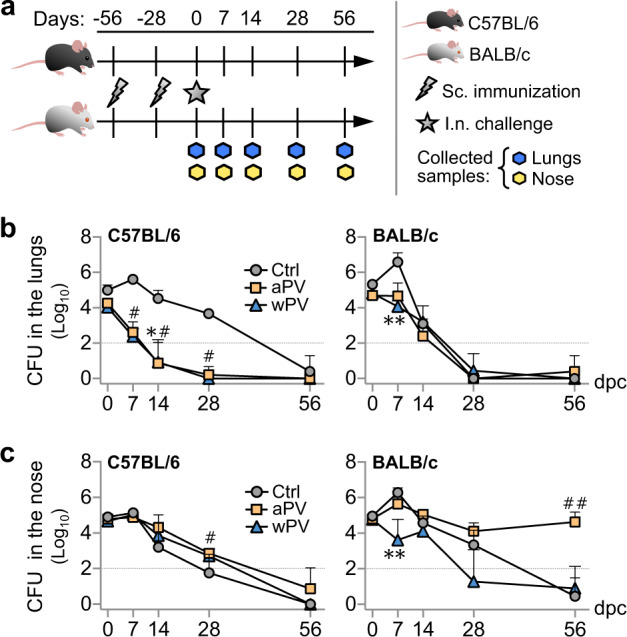
aPV prolongs nasal carriage of Bp in BALB/c mice. **a** Schematic of vaccination and challenge protocol. Six-week-old C57BL/6 and BALB/c mice were immunized twice subcutaneously (s.c.) with 1/10 human dose of Infanrix (aPV) or Shan5 (wPV) vaccine at a 4-week interval or left unvaccinated (Ctrl) and then nasally (i.n.) challenged with 10^6^ CFU of B1917GR 4 weeks after the second immunization. Bacterial burden in the noses and lungs was determined by CFU counting at indicated time points post challenge on homogenized organs. **b** CFU counts on lung homogenates at indicated time points. **c** CFU counts on nose homogenates at indicated time points. Results shown are geometric means ± SD. *n* = 4–5. Kruskal–Wallis tests were performed to compare aPV- (^#^) and wPV- (*) immunized mice to controls. Only significant differences are indicated. *^,^^#^*P* < 0.05, **^,##^*p* < 0.001, ***^,###^*p* < 0.001, and ****p < 0.0001. Dashed lines in CFU counts correspond to the detection limit.

To evaluate the relative protection conferred by wPV and aPV in the upper and lower respiratory tracts of mice, 6-week-old C57BL/6 and BALB/c mice were immunized twice subcutaneously with 1/10 human dose of Infanrix (aPV) or Shan5 (wPV) vaccine at a 4-week interval or left unvaccinated as controls (Fig. 1a) and challenged intranasally 4 weeks after the second immunization with B1917GR, a gentamycin-resistant derivative of a clinical Bp strain representative of currently circulating strains^23^. The bacterial burden in the noses and lungs of the mice was determined by counting colony-forming units (CFUs) on the homogenized organs at different times post challenge (p.c.) (3 hours p.c. (h.p.c.) (D0) and 7, 14, 28, 56 days p.c. (d.p.c.)) (Fig. 1a).

Both aPV and wPV protected equally well against lung colonization after infection with a high-dose challenge (10^6^ CFU) (Fig. 1b). Protection was also evident when the mice were infected with a lower dose (10^5^ CFU) (Supplementary Fig. 1a). However, neither vaccine protected against nasal infection of C57BL/6 mice (Fig. 1c, left panel), even when the mice were challenged with a moderate dose of Bp (Supplementary Fig. 1b, left panel). In BALB/c mice, immunization with wPV led to a slight, but significant reduction of nasal colonization at the early time points after infection, whereas aPV provided no protection at all (Fig. 1c, right panel and Supplementary Fig. 1b, right panel). Instead, vaccination with aPV resulted in significantly prolonged nasal carriage. The amounts of CFU in the nasal tissues were as high at 56 d.p.c. as they were in the beginning of the infection, with no trend of a decrease in bacterial load (Fig. 1c, right panel and Supplementary Fig. 1b, right panel).

### Both BALB/c and C57BL/6 mice develop resident memory Th17 cells in the nose after Bp infection

**Fig. 2:**
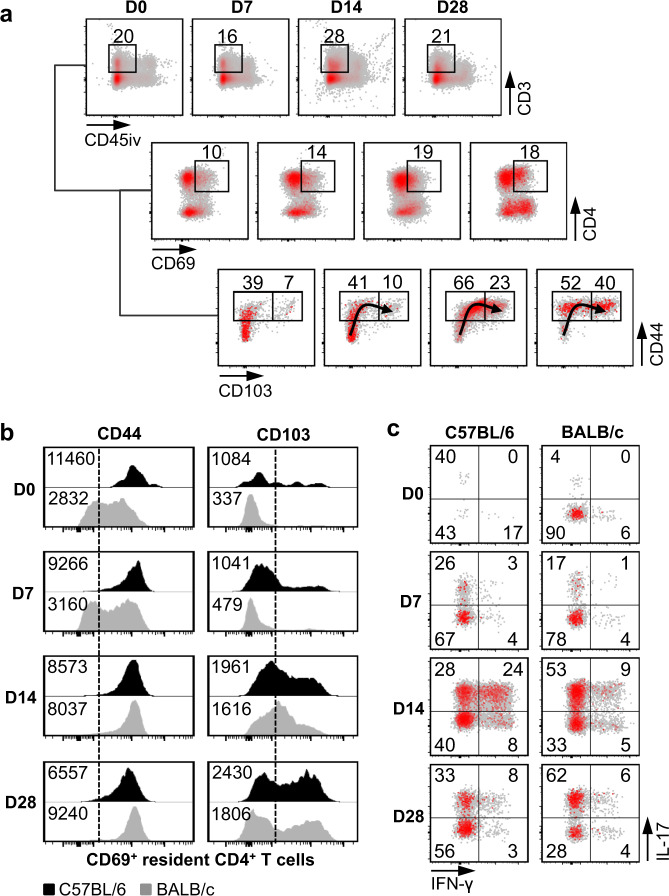
Bp-infected BALB/c mice develop Th17 T_RM_ cells in the nose. Six-week-old C57BL/6 mice and BALB/c mice were infected with 10^6^ CFU of B1917GR. Nasal immune cells were collected at indicated time points post challenge (D0–D28). Ten minutes before euthanasia, mice were intravenously injected with anti-CD45-PE antibody enabling us to discriminate T_RM_ cells from circulating cells. T_RM_ cells express CD44, CD69, and/or CD103. Cell activation was assessed by nonspecific ex vivo stimulation and intracellular staining of IL-17 and IFN-γ. Cells were fixed and permeabilized to perform intracellular staining of IL-17 and IFN-γ. Numbers indicate percentages of events in each square. **a** Representative graph showing the accumulation of CD103^+^ CD44^+^ T_RM_ cells in the nose of BALB/c mice at indicated time points after Bp challenge. **b** Representative graph comparing the pattern of expression of CD44 (left panels) and CD103 (right panels) in the nose of C57BL/6 (black) and BALB/c mice (gray) at indicated time points after Bp challenge. Numbers indicate mean fluorescence intensities and dotted lines indicate the cut-off between positive and negative cells. **c** Representative graph showing the proportion of IL-17- and/or IFN-γ-producing CD4^+^ T_RM_ cells in the nose of C57BL/6 (left panels) and BALB/c mice (right panels) at indicated time points after Bp challenge. Numbers indicate percentages of events in each square.

IL-17-producing CD4^+^ T_RM_ cells, expressing CD69, CD44, and/or CD103, were recently shown to correlate with protection against nasal colonization by Bp in C57BL/6 mice^20,24^. We, therefore, investigated whether the Th2-prone BALB/c mice might be less efficient than C57BL/6 mice in inducing these cells in the nasal tissues upon Bp infection. To identify T_RM_ cells in the nasal tissues, mice were injected intravenously with an anti-CD45-PE antibody (CD45iv), enabling us to distinguish infiltrated resident cells from circulating immune cells. Following the intranasal challenge, up to ~30% of immune cells in the noses of BALB/c mice were lymphocytes expressing CD3 (Fig. 2a). Among the CD3^+^ T cells, the proportion of CD69^+^ CD4^+^ T cells doubled 14 d.p.c. (Fig. 2a). These CD69^+^ CD4^+^ T cells up-regulated CD44 and CD103 expression over time after infection (Fig. 2a), indicating that BALB/c mice were able to induce T_RM_ cells in the nasal tissue after Bp infection. However, compared to C57BL/6 mice, expression of these T_RM_ markers was somewhat delayed in BALB/c mice (Fig. 2b). Nonetheless, at 14 d.p.c. C57BL/6 and BALB/c mice shared a similar expression pattern of both markers (Fig. 2b).

Measurement of IL-17- and/or IFN-γ-producing cells after stimulation with phorbol myristate acetate (PMA) and ionomycin in the presence of brefeldin A showed that similar to T_RM_ cells from C57BL/6 mice, T_RM_ cells from BALB/c mice were activated quickly after challenge and expressed mainly IL-17 (Fig. 2c). However, only 14% of T_RM_ cells from BALB/c mice versus 32% of T_RM_ cells from C57BL/6 mice expressed IFN-γ or both IL-17 and IFN-γ 14 d.p.c. (Fig. 2c). Thus, BALB/c mice are not intrinsically unable to induce nasal IL-17-producing CD4^+^ T_RM_ cells upon infection with Bp.

### aPV immunization impairs the induction of nasal Th1 and Th17 T_RM_ cells in BALB/c mice upon Bp infection

**Fig. 3:**
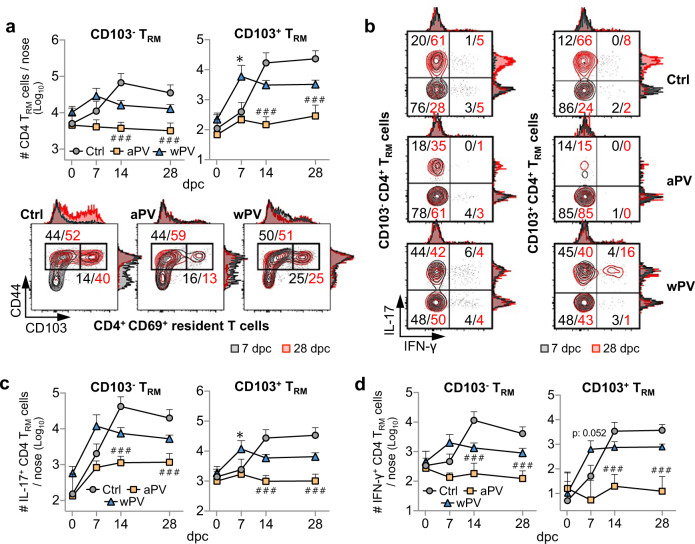
aPV immunization impairs TRM cell induction in the noses of BALB/c mice. BALB/c mice were immunized twice with aPV or wPV or left unvaccinated (Ctrl) prior to challenge with 10^6^ CFU of B1917GR and the induction of CD4^+^ T_RM_ cells in the noses of mice was measured at the indicated time points. **a** Absolute numbers of nasal CD103^−^ (upper left panel) and CD103^+^ (upper right panel) CD4^+^ T_RM_ cells and representative graph illustrating differential enrichment of CD44^+^ CD103^+^ among the CD69^+^ CD4^+^ resident T cells 7 (in black) and 28 d.p.c. (in red) in the nose of control and vaccinated BALB/c mice (lower panels). Numbers indicate percentages of events in each square. **b** Representative graphs showing the increase in IL-17- and/or IFN-γ-producing nasal CD103^−^ (left panels) and CD103^+^ (right panels) CD4^+^ T_RM_ cells between 7 (in black) and 28 d.p.c. (in red). Numbers indicate percentages of events in each square. **c** Absolute numbers of IL-17^+^ CD103^−^ (left panel) and IL-17^+^ CD103^+^ (right panel) nasal CD4^+^ T_RM_ cells at indicated time points after Bp challenge. **d** Absolute numbers of nasal IFN-γ^+^ CD103^−^ (left panel) and IFN-γ^+^ CD103^+^ (right panel) CD4^+^ T_RM_ cells at indicated time points after Bp challenge. Results shown are geometric means ± SD. *n* = 6 per group. Kruskal–Wallis tests were performed to compare aPV (^#^) and wPV (*) immunized mice to control mice. Only significant differences are indicated. *P < 0.05; ^###^*p* < 0.001.

As Bp infection leads to the induction of Th1 and Th17 T_RM_ cells in the nasal tissues of BALB/c mice, we examined the effect of aPV or wPV immunization on Bp infection-induced T_RM_ cells in the noses of these mice. Whereas upon Bp infection an increase in both CD103^+^ CD44^+^ and CD103^−^ CD44^+^ T_RM_ cells was detectable among CD69^+^ CD4^+^ T cells in the nasal tissues of BALB/c mice from 7 d.p.c. on and peaking at 14 d.p.c., prior aPV vaccination completely abolished the induction of these cells (Fig. 3a, upper panels). Representative examples of CD103^+^ CD44^+^ T cell enrichment among CD69^+^ CD4^+^-resident T cells between 7 and 28 d.p.c. are shown in Fig. 3a (lower panels). In contrast, immunization with wPV did not inhibit the induction of CD4^+^ T_RM_ cells upon Bp infection, but instead resulted in earlier induction of the CD103^+^ CD44^+^ T_RM_ cells that were significantly enriched 7 d.p.c. compared to non-vaccinated mice (Fig. 3a).

In addition, the CD103^+^ CD4^+^ T_RM_ cells from aPV-immunized mice were poorly activated, as <20% of CD103^+^ T_RM_ cells from aPV-immunized mice expressed IL-17 or IFN-γ upon stimulation with PMA and ionomycin 7 or 28 d.p.c., whereas most of (>50%) the stimulated CD103^+^ CD4^+^ T_RM_ cells from control and wPV-immunized mice produced IL-17 and/or IFN-γ 28 d.p.c. (Fig. 3b). IL-17^+^IFN-γ^+^ cells were also enriched in the CD103^+^ CD4^+^ T_RM_ cell subset of control and wPV-immunized mice. No IL-17^+^IFN-γ^+^ cells were detected in aPV-immunized mice. Absolute counts of IL-17^+^ T_RM_ and IFN-γ^+^ T_RM_ cells show that IL-17^+^ T_RM_ cells predominated in wPV-immunized mice (Fig. 3c, d). IL-17^+^ CD103^+^ T_RM_ cells were significantly enriched in wPV-immunized mice compared to control mice 7 d.p.c., while at later time points after infection, the numbers of IL-17^+^ CD103^+^ T_RM_ cells tended to be higher in control mice than in wPV-immunized mice (Fig. 3c). A similar observation was made for the IFN-γ-producing CD103^+^ CD4^+^ T_RM_ cells (Fig. 3d). Thus, early protection conferred by wPV against nasal colonization of BALB/c mice was associated with a rapid expansion of IL-17^+^ CD103^+^ CD4^+^ T_RM_ and to a lesser extend IFN-γ^+^ CD103^+^ CD4^+^ T_RM_ cells. In contrast to wPV, aPV immunization totally inhibited the induction of these cells in the nasal tissues of Bp-infected BALB/c mice. A similar trend was also observed for IL-17^+^ CD103^−^ CD4^+^ T_RM_ and for IFN-γ^+^ CD103^−^ CD4^+^ T_RM_ cells.

To examine whether the difference between the aPV and wPV effect on the inhibition of T_RM_ cell induction in the nose of BALB/c mice might be related to differences in serum antibody responses to the two vaccines, we measured serum IgG titers to whole *B. pertussis* cell extracts in aPV- and in wPV-immunized mice before and after *B. pertussis* challenge. Both before and after the challenge, wPV and aPV induced high levels of anti-*B. pertussis* serum antibodies (Supplementary Fig. 2). These titers were slightly, but significantly higher in wPV-immunized than in aPV-immunized mice, making it unlikely that the difference in nasal T_RM_ cell induction was due to a blunting effect by aPV-induced serum antibodies.

### IL-17 is essential for the control of nasal but not of lung infection by Bp

**Fig. 4:**
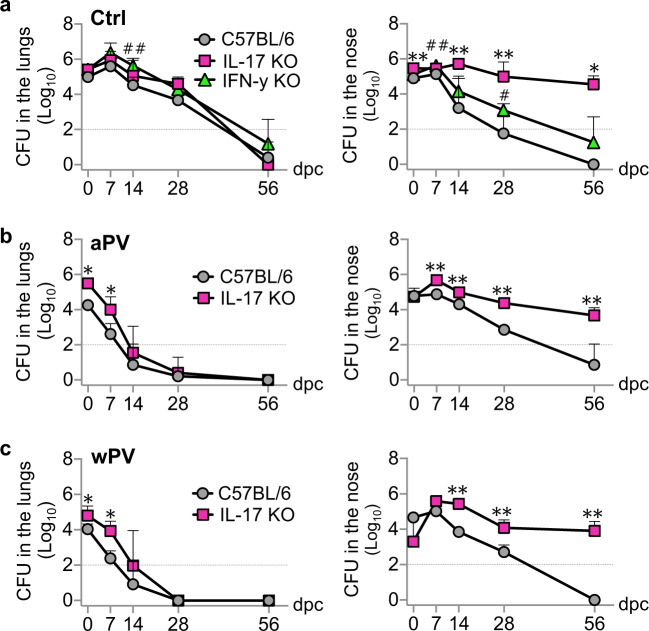
IL-17 is required for the control of nasal colonization by Bp. **a** C57BL/6 (in gray), IL-17 KO (in purple), and IFN-γ KO (in green) mice were infected with 10^6^ CFU of B1917GR, and CFU numbers were counted in lungs (left panel) and noses (right panel) at indicated time points. **b** C57BL/6 (in gray), IL-17 KO (in purple) mice were immunized with aPV and then infected with 10^6^ CFU of B1917GR, and CFU numbers were counted in lungs (left panel) and noses (right panel) at indicated time points. **c** C57BL/6 (in gray), IL-17 KO (in purple) mice were immunized with wPV and then infected with 10^6^ CFU of B1917GR, and CFU numbers were counted in lungs (left panel) and noses (right panel) at indicated time points. *n* = 3–5. Mann–Whitney tests were performed to compare C57BL/6 mice and IL-17 (*) or IFN-γ (^#^) knockout mice. *P < 0.05, **p < 0.01, and ^##^*p* < 0.01. Only significant differences are indicated. Dashed lines correspond to the detection limit.

As aPV immunization appeared to prevent natural clearance of nasal Bp colonization and to inhibit the induction of IL-17- and/or IFN-γ-producing CD4^+^ T_RM_ in the noses of Bp-infected BALB/c mice, we investigated the relative role of IL-17 and IFN-γ in controlling nasal carriage of Bp. IL-17 knockout (KO) mice and IFN-γ KO mice were infected nasally with Bp, and lung and nasal colonization was followed over time. Whereas both KO mice cleared the lung infection as quickly as the control mice, regardless of the initial bacterial burden (Fig. 4a, left panel and Supplementary Fig. 3a, left panel), IL-17-deficient mice were unable to control nasal infection by Bp and carried the infection for at least 56 d.p.c. without a trend of decrease in bacterial load (Fig. 4a, right panel and Supplementary Fig. 3a, right panel). IFN-γ deficiency only slightly affected Bp clearance compared to control mice.

We then assessed the effect of aPV and wPV immunization on lung and nasal clearance of Bp in IL-17 KO mice compared to control mice. With a moderate challenge dose, either vaccine provided protection against lung colonization in IL-17 KO mice similar to the control mice (Supplementary Fig. 3b, c, left panels, respectively), while protection conferred by aPV and wPV was slightly impaired in IL-17 KO mice when mice were infected with a high inoculum (Fig. 4b, left panel and Fig. 4c, left panel, respectively). As expected, neither vaccine was able to provide protection against nasal carriage of the IL-17 KO mice, regardless of the challenge dose (Fig. 4b, c, right panels and Supplementary Fig. 3b, c, right panels).

### T_RM_ cell-mediated protection against nasal colonization depends on IL-17

**Fig. 5:**
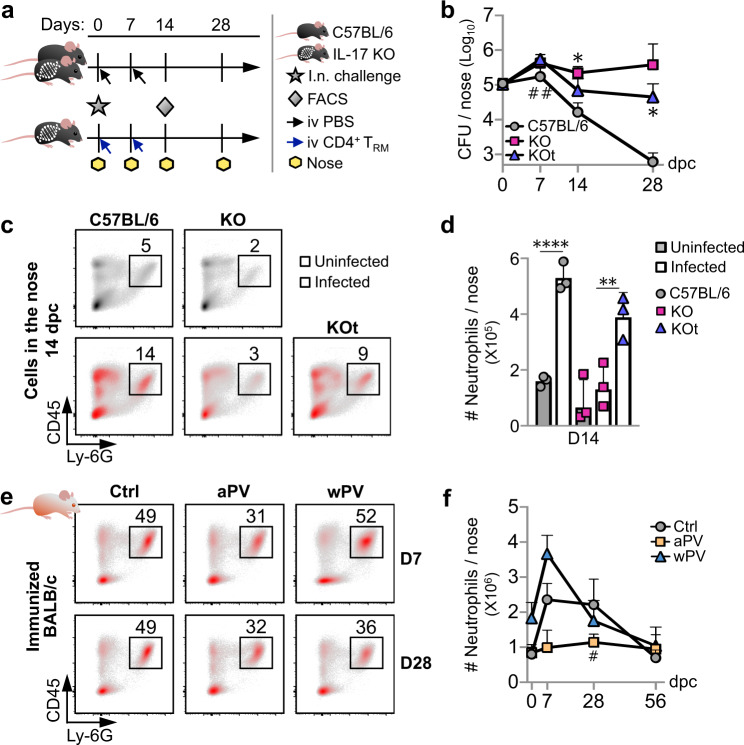
Bp–specific nasal CD4^+^ T_RM_ cells protect IL-17 KO mice against nasal colonization and induce recruitment of neutrophils. **a** Schematic of the transfer protocol. C57BL/6 mice were infected nasally (i.n.) with 10^6^ CFU of B1917GR. CD4^+^ T_RM_ cells were purified from noses of infected C57BL/6 mice 14 d.p.c. A total of 10^4^ CD45iv^−^ CD45^+^ CD44^+^ CD69^+^ CD4^+^ T_RM_ cells were transferred intravenously (i.v.) to IL-17 KO mice 10 h.p.c. and 7 d.p.c. (blue arrows). Control IL-17 KO and C57BL/6 mice received PBS (black arrows). CFU in the nasal tissues was counted at indicated time points post challenge. Nasal cells were collected 14 d.p.c. and analyzed by flow cytometry (FACS). **b** C57BL/6 (in gray), IL-17 KO (KO, in purple), and transferred IL-17 KO mice (KOt, in blue) were infected with 10^5^ CFU of B1917GR, and CFU numbers were counted in the noses at indicated time points. **c** Representative graphs showing neutrophil (CD45iv^−^ CD45^+^ Ly-6G^+^) recruitment in the nose of C57BL/6 (left panels), IL-17 KO (KO, middle panels), and transferred IL-17 KO mice (KOt, right panel) mice 14 days after infection with 10^5^ CFU of B1917GR. Neutrophil recruitments of non-infected and infected mice are depicted in the upper (gray) and lower (red) panels, respectively. Numbers indicate percentages of events in each square. **d** C57BL/6 (in gray), IL-17 KO (KO, in purple), and transferred IL-17 KO mice (KOt, in blue) mice were infected with 10^5^ CFU of B1917GR (open bars) or left uninfected (gray bars), and absolute numbers of neutrophils per nose were measured 14 d.p.c. **e** Representative graphs showing neutrophil (CD45^+^ Ly-6G^+^) recruitment in the nose of BALB/c mice vaccinated with aPV (middle panels) or wPV (right panels) or left unvaccinated (left panels) 7 days (7 d.p.c.) and 28 days (28 d.p.c.) after infection with 10^6^ CFU of B1917GR. Numbers indicate percentages of events in each square. **f** Absolute numbers of neutrophils in the noses of BALB/c mice immunized with aPV (in yellow) or wPV (in blue), or left un-immunized (in gray) at indicated time points after challenge with 10^6^ CFU of B1917GR. Results shown are geometric means ± SD. *n* = 3–5. Ordinary one-way ANOVA (**b**, **d**) were performed to compare KO mice to KOt (*, **b**, **d**) or to C57BL/6 mice (^#^, **b**) and to evaluate the induction of neutrophils in infected C57BL/6 (*, **d**). Kruskal–Wallis tests were performed to compare aPV (^#^) and wPV (*) immunized BALB/c mice to control mice (**f**). *^,#^*P* < 0.05, **^,##^*p* < 0.001, ***p < 0.001, and ****p < 0.0001. Only significant differences are indicated.

As IL-17 appeared to be essential for nasal clearance of Bp infection, we assessed whether transfer of IL-17^+^ CD103^+^ CD4^+^ T_RM_ cells could rescue the ability of IL-17 KO mice to clear nasal infection by Bp. A total of 10^4^ CD4^+^ T_RM_ cells isolated from the noses of C57BL/6 mice 14 d.p.c. were transferred intravenously (i.v.) to IL-17 KO mice (KOt), 10 h.p.c. and 7 d.p.c. (Fig. 5a). Control mice were injected intravenously with phosphate-buffered saline (PBS). CFUs in the noses were counted 3 h.p.c. and 7, 14, and 28 d.p.c. The first transfer of CD4^+^ T_RM_ cells did not affect nasal colonization compared to non-transferred mice, as at 7 d.p.c. the bacterial load in the noses of KOt and KO mice were similar (Fig. 5b). After the second transfer given 7 d.p.c., the bacterial load in the noses of KOt mice started to decrease at a rate similar to that of C57BL/6 control mice, indicating that the transfer of Bp-induced CD4^+^ T_RM_ cells allowed IL-17 KO mice to reduce nasal Bp infection as efficiently as infected C57BL/6 mice (Fig. 5b).

### Lack of CD4^+^ T_RM_ cell induction results in lack of neutrophil recruitment

In an attempt to identify the effector cells involved in nasal clearance, we examined the nasal influx of neutrophils upon Bp infection. CD45^+^ Ly-6G^+^ neutrophils infiltrated in the noses of Bp-infected C57BL/6 mice 14 d.p.c. were compared to non-infected mice (Fig. 5c, d). No significant increase in neutrophils was observed in infected IL-17 KO mice, whereas transfer of nasal CD4^+^ T_RM_ cells from Bp-infected C57BL/6 mice resulted in significant neutrophil recruitment in the noses of IL-17 KO mice 14 d.p.c. (Fig. 5c, d). The decrease in nasal bacterial loads in C57BL/6 and KOt mice over non-transferred IL-17 KO mice was thus associated with an increase in the amounts of recruited neutrophils (Fig. 5d). Overall, the data suggest that upon Bp infection nasal CD4^+^ T_RM_ cells induce the recruitment of neutrophils by releasing IL-17 and thereby enhance bacterial clearance.

To determine whether the inability of aPV-immunized BALB/c mice to clear the nasal infection by Bp was also due to their failure in recruiting neutrophils in the noses, we immunized BALB/c mice with aPV or wPV and examined the recruitment of nasal neutrophils over time. While Bp infection of non-vaccinated BALB/c mice resulted in a significant increase in the nasal influx of neutrophils, this influx was totally abolished in aPV-immunized, but not in wPV-immunized mice (Fig. 5e, f). The recruitment of other nasal CD3^−^ CD11b^+^ immune cells was not impaired in aPV-immunized mice (Supplementary Fig. 4).

### aPV immunization allows extracellular persistence of Bp in the noses of BALB/c mice

**Fig. 6:**
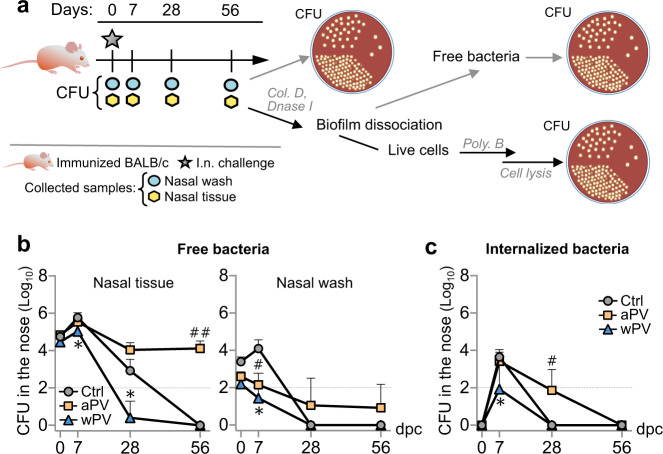
aPV immunization prevents clearance of extracellular, tightly adhering Bp in the noses of BALB/c mice. **a** Schematic of experimental procedures. aPV- (in yellow in **b**, **c**), wPV-immunized (in blue in **b**, **c**) or control BALB/c mice (Ctrl, in gray in **b**, **c**) were challenged with 10^6^ CFU of B1917GR and euthanized at indicated time points post challenge. NWs were collected before nose harvest to count extracellular CFU in the nasal fluids. Nasal tissue was digested with collagenase D (col. D) and DNase I for biofilm dissociation, flushed, and passed through a cell strainer to obtain a single-cell suspension. Cells were pelleted and extracellular CFUs were counted. Cells were incubated with polymyxin B (poly. B), washed and lysed to estimate the number of intracellular CFU. **b** Extracellular CFU in the nasal tissues (left panel) and nasal washes (right panel). **c** Intracellular CFU in the nasal tissue. Results shown are geometric means ± SD. *n* = 3–5. Kruskal–Wallis tests were performed to compare aPV- and wPV-immunized mice to control mice. *^,#^*P* < 0.05; **^,##^*p* < 0.01. Only significant differences are indicated. Dashed lines in **b**, **c** correspond to the detection limit.

As in vitro data suggest that Bp can invade and survive within eukaryotic cells^25^ and can also adopt a biofilm lifestyle allowing persistent colonization in mice^26^, we assessed the impact of aPV immunization on intracellular versus extracellular nasal Bp carriage. aPV- or wPV-immunized BALB/c mice were nasally infected with Bp B1917GR, and nasal washes (NWs) and homogenates were assessed for CFU counts at different time points after challenge (Fig. 6a). The nasal homogenates were treated with collagenase D and DNase I for tissue and biofilm disruption^26,27^. After treatment, the cells were harvested by centrifugation and extracellular bacteria were counted by CFU measurements of the supernatants. To measure the amounts of intracellular bacteria, the cells were washed and incubated with polymyxin B in order to kill potentially remaining extracellular bacteria. Cells were then lysed and intracellular bacteria were quantified by CFU measurements.

Approximately 10- to 100-fold more bacteria were found in the nasal homogenates compared to the NWs at each time point, suggesting that most bacteria were tightly associated with the nasal epithelium (Fig. 6b). In contrast to control mice and wPV-immunized mice, the bacteria remained associated with the nasal epithelium in aPV-immunized mice for at least up to 56 d.p.c., whereas both vaccines reduced the bacterial load in the NWs (Fig. 6b). Virtually no intracellular bacteria were detected 3 h.p.c. (day 0), whereas substantial amounts of intracellular bacteria were detectable at 7 d.p.c., especially in control mice and in aPV-immunized mice (Fig. 6c). Intracellular bacteria were eventually cleared in all three groups 28 d.p.c. (Fig. 6c).

## Discussion

Several reasons have been proposed to explain the resurgence of pertussis, especially in countries using aPV, although many studies have shown a good efficacy of aPV against pertussis disease. These reasons include the rapid waning of protective immunity conferred by aPV^6,28^ compared to wPV^29,30^ and the inability of aPV, as well as of wPV to prevent Bp infection and pertussis transmission. Using mouse models, we show here that in addition to the failure of immunization with aPV or wPV to protect against nasal colonization aPV immunization resulted in substantially prolonged nasal Bp carriage in Th2-prone BALB/c mice when compared to non-immunized mice, while it was protective against lung colonization. These findings are consistent with observations in a non-human primate model showing that aPV immunization, while protecting against disease, caused prolonged carriage of Bp-challenged baboons^8^. We found that aPV-induced prolonged nasal carriage in BALB/c mice was paralleled with vaccine-mediated inhibition of nasal IL-17- and/or IFN-γ-producing CD103^+^ CD4^+^ T_RM_ cells induced by Bp infection. Recently, Wilk et al.^20^ showed that, in contrast to wPV immunization, aPV immunization fails to expand T_RM_ cells in the lungs and noses and provided evidence that T_RM_ cells promote bacterial clearance in mouse lungs by adoptive transfer of CD4^+^ T_RM_ cells in irradiated mice or by treating mice with FTY720 during the immunization phase, which prevents migration of T and B cells from lymph nodes to the circulation^19,20^. We found here that aPV not only fails to induce IL-17^+^ and/or IFN-γ^+^ CD4^+^ T_RM_ cells, but even prevents the induction of these cells in the nasal tissue of Bp-infected BALB/c mice, while they were strongly induced in non-vaccinated mice upon Bp challenge, peaking at 14 d.p.c., and were maintained for at least up to 28 d.p.c. In contrast, in wPV-immunized mice, these cells were induced earlier than in non-vaccinated mice, and peaked at 7 d.p.c., which was paralleled by a significant decrease in bacterial burden in the noses of wPV-immunized BALB/c mice 7 d.p.c. compared to non-vaccinated controls.

While Bp infection resulted in the expansion of both IFN-γ-producing Th1 and IL-17-producing Th17 CD4^+^ T_RM_ cells in the nasal tissue, the latter were the main cells involved in the control of nasal colonization by Bp, as IL-17 KO mice were unable to clear nasal Bp infection, while able to control lung infection as well as control mice. In contrast, IFN-γ KO mice cleared the infection both in lungs and noses, similar to control mice. Furthermore, adoptive cell transfer of nasal CD4^+^ T_RM_ cells isolated from Bp-infected C57BL/6 mice to IL-17-deficient mice conferred protection against nasal colonization. Thus, aPV immunization prevents nasal Bp clearance in Th2-prone mice by preventing the natural induction of nasal IL-17^+^ CD103^+^ CD4^+^ T_RM_ cells upon Bp infection.

IL-17 deficiency was associated with failure to recruit neutrophils upon Bp infection, and adoptive transfer of Th17 T_RM_ cells to IL-17 KO mice was associated with accelerated neutrophil recruitment, suggesting that Th17 T_RM_ cells control the outcome of the infection by enhancing neutrophil recruitment in the noses of Bp-infected mice. Through releasing of IL-17, Th17 T cells are known to attract neutrophils to the site of infection via CXC chemokines and to modulate neutrophil granulopoiesis via the induction of endogenous granulocyte colony-stimulating factor and stem cell factor^31,32^. Consistent with a protective role of neutrophils in immunized or convalescent mice, neutropenia severely impairs clearance^33^. Furthermore, Bp clearance from mouse lungs after the transfer of antibodies from convalescent mice was abrogated by neutrophil depletion or Fcγ receptor deletion^34^, suggesting that neutrophils mediate the killing of Bp in the lungs by opsonic phagocytosis. We recently showed that passive transfer of serum from aPV-immunized mice or mice immunized with the live attenuated BPZE1 vaccine conferred protection in the lungs of severe combined immunodeficiency mice but not in the nose, suggesting that serum immunoglobulins (Igs) do not efficiently protect in the nose^21^. Instead, by using knockout models and passive transfer of mucosal Igs, it was demonstrated that mucosal IgA induced by BPZE1 contributes to nasal protection as well as IL-17, possibly through the induction of Th17 T_RM_ cells.

We found that Bp was present in three locations in the noses of BALB/c mice. A minority was loosely attached to the nasal epithelium and could easily be recovered in NWs. A small fraction was found in intracellular compartments and the vast majority of Bp organisms was tightly attached to the nasal epithelium. Both wPV and aPV immunization diminished the loosely attached bacteria, suggesting that they may have been cleared by transuded wPV- or aPV-induced serum antibodies. Immunization with aPV slightly prolonged the presence of intracellular bacteria in the noses of BALB/c mice. The strongest effect of aPV immunization was seen on the Bp organisms that were tightly attached to the nasal epithelium, suggesting a major role of IL-17^+^ T_RM_ cells in clearing tightly attached bacteria. IL-17 plays a central role in maintaining the mucosal barrier integrity^35,36^, and Bp has been shown to alter the epithelial cell barrier^37^, which results in the exposure of cryptic receptors on the basement membrane allowing the pathogen to attach more efficiently^38^.

The localization of CD103^+^ CD4^+^ T_RM_ cells in the nasal epithelium positions them well to act as the first line of defense at subsequent exposure to Bp. While CD103^+^ T_RM_ cells interact with epithelial cells through E-cadherin^39,40^, CD44^+^ T cells may be retained in the underlying extracellular matrix^41^. The greatest differences in T_RM_ cell numbers and activation between aPV-immunized BALB/c mice and control mice were observed in the CD103^+^ subset of T_RM_ cells. In addition, early accumulation of CD103^+^ T_RM_ cells by wP-primed mice compared to control mice was associated with a decrease in nasal bacterial burden at early time points after Bp infection, thereby confirming the importance of these cells to clear bacteria in the nose. Augmented expression of IL-17/IFN-γ double-positive cells in the CD103^+^ T_RM_ cell subset may enhance local immune responses in the nasal mucosa, as these cytokines are known to enhance phagocytosis. In addition, IL-17/IFN-γ double-positive cells have been shown to induce the expression of antimicrobial genes by epithelial cells, further suggesting a protective role of CD103^+^ T_RM_ cells^42^.

In conclusion, we provide evidence here that aPV immunization may prolong nasal carriage of Bp and thereby increase the Bp reservoir in aPV-immunized populations, which may have contributed to the increase in pertussis incidence in countries in which aPV are in use. The effect of aPV on the prolonged nasal carriage was seen in Th2-prone BALB/c mice, which were unable to clear nasal Bp carriage, especially Bp organisms that tightly adhere to the nasal epithelium, for at least up to 56 d.p.c. These findings are relevant in the context of newborns in which a bias toward a Th2-type differentiation has been demonstrated^22^, but may also be relevant for older Th2-prone individuals. The aPV effect on nasal carriage is due to aPV-mediated inhibition of IL-17^+^ CD103^+^ CD4^+^ T_RM_ cells expansion, which in turn prevents neutrophil recruitment important for bacterial clearance. This study provides thus a coherent explanation of how aPV enhances nasal carriage of Bp and supports the hypothesis of asymptomatic Bp carriage and transmission as a major driver of pertussis resurgence in countries that have switched from wPV to aPV immunization programs^9^. However, aPV induces antibodies that provide protection against lung colonization in mice and against pertussis disease in humans, which is the purpose of these vaccines.

## Methods

### Bacterial strain

Bp strain B1917, a pertactin-producing strain, came from the RIVM collection (Bilthoven, The Netherlands). For counter selection purposes, B1917 was electroporated with the pFUS2-BctA1 suicide plasmid to acquire gentamicin resistance as described^43^, thereby yielding B1917GR. After electroporation, gentamicin-resistant derivatives were checked by PCR to verify the site of insertion of the pFUS2-BctA1 suicide vector in the bacterial genome. Bacteria were cultured at 37 °C on Bordet-Gengou (BG) agar (Difco Bordet-Gengou Agar Base), supplemented with 1% glycerol, 10% defibrinated sheep blood, and 10 μg/ml gentamycin, as described^44^. After 40 h of growth, the bacteria were harvested by scraping the plates and resuspended in PBS at the density of 5 × 10^7^ or 5 × 10^6^ CFU/ml. Whole *B. pertussis* cell lysates were prepared and used for antibody determination as described^45^.

### Animals and ethical statement

BALB/c and C57BL/6 mice were purchased from Charles Rivers France. IL-17 and IFN-γ KO mice were kindly provided by F. Trottein (Institut Pasteur de Lille). All the animal experiments were carried out in accordance with the guidelines of the French Ministry of Research, and the protocols were approved by the Ethical Committees of the Region Nord Pas de Calais and the Ministry of Research (agreement number APAFIS # 2019052015506229 V3).

### Protection experiments

Mice were immunized subcutaneously with 1/10 human dose of aPV (Infanrix, GlaxoSmithKline) or wPV (Shan5, Shantha Biotechnics, India) and boosted 4 weeks later with the same dose. Control mice were injected with PBS. Eight weeks after the first immunization, mice were challenged intranasally with 10^6^ or 10^5^ CFU of virulent B1917GR. Mice were euthanized at different times post challenge, that is 3 h.p.c. and 7, 14, 28, and 56 d.p.c. NWs were performed with stainless steel feeding tubes (24 ga × 25 mm, Phymep). One milliliter of PBS was slowly injected through the trachea and the NW was collected. Lungs and noses were harvested and homogenized for CFU measurement by plating 10-fold serial dilutions onto BG agar plates containing 10 μg/ml gentamicin as described^21^.

### Cell isolation for CFU tests

Infected BALB/c mice were euthanized at different times post challenge (3 h.p.c. and 7, 28, and 56 d.p.c.). NWs were collected prior to nose harvest to assess extracellular CFU in the nasal fluid. Nasal tissue was scraped from the nasal cavity, cut into small pieces, and digested with collagenase D (0.6 mg/ml; Roche) and DNase I (20 U/ml; Sigma-Aldrich) for 40 min at 37 °C. The nasal tissue was flushed several times and then passed through a cell strainer to obtain a single-cell suspension. Cells were pelleted and the supernatant was conserved to assess extracellular CFU. Cells were washed twice and then incubated for 1 h with 100 µg/ml polymyxin B sulfate (Sigma) to eliminate extracellular bacteria^46^. Cells were washed twice and then lysed with 0.1% saponin (Sigma). CFU counting was performed on cell lysates by plating 10-fold serial dilutions onto BG agar plates containing 10 μg/ml gentamicin to estimate the number of intracellular bacteria.

### Cell isolation for flow cytometry analysis

Ten minutes before euthanasia, mice were injected intravenously with 10 μg of anti-CD45-PE antibody from eBioscience (30-F11, catalog#12-0451-82) to allow for the distinction of circulating T cells (CD45-PE^+^) and resident T cells or infiltrated immune cells (CD45-PE^−^). Nasal tissue was treated as described above. Red blood cells were lysed using ACK lysing buffer (Gibco). Isolated cells were stimulated with 50 ng/ml phorbol myristate (InVivoGen) and 500 ng/ml ionomycin (Sigma) in the presence of 5 µg/ml brefeldin A (Sigma) for 4 h at 37 °C. Cells were incubated with Dead Cell Stain Kit LIVE/DEAD Aqua (Invitrogen) first as recommended by the supplier, then with Fc block (BD Biosciences, dilution 1:50), followed by surface staining with the following fluorochrome-conjugated antibodies (dilution 1:400): CD45-APC (30-F11, catalog#559864), CD69-FITC (H1.2F3, catalog#11-0691-82), CD8-AF700 (53-6.7, catalog#56-0081-82), CD3-APC-eF780 (17A2, catalog#47-0032-82) from eBioscience, CD44-BV605 (IM7, catalog#103047), CD4-BV785 (RM4-5, catalog#100552) from BioLegend, and CD103-PE-CF594 (M290, catalog#565849) from BD Biosciences. For the detection of intracellular cytokines, cells were fixed and permeabilized using a FoxP3 transcription factor staining buffer set (eBioscience), incubated with normal rat serum (eBioscience) (1:50), and stained with the following antibodies: IL-17-V450 (TC11-18H10, catalog#560522, dilution 1:200) from BD Biosciences and IFN-γ-PE-Cy7 (XMG1.2, catalog#25-7319-41, dilution 1:400) from eBioscience. Fluorescence minus one sample were used as controls. The gating strategy for the identification of IL-17^+^ T_RM_ cells is shown in Supplementary Fig. 5a. Fluorescence-activated cell sorting samples were acquired on an LSR Fortessa using the BD Diva Software (BD Biosciences) and analyzed using the FlowJo Software (v10, TreeStar). Neutrophils were identified with Ly-6G-PE-CF594 (1A8, catalog#562700, dilution 1:400) and CD11b-BV605 (M1/70, catalog#563015, dilution 1:400) antibodies from BD Biosciences (see Supplementary Fig. 5b). The total number of cells was calculated by multiplying the number of registered events by the ratio of CD45-APC^+^ cells over the absolute number of immune cells in the nasal tissue, determined by counting live immune cells in a hemocytometer.

### Adoptive transfer of CD4^+^ T_RM_ cells from the nose

CD4^+^ T_RM_ cells were purified from noses of C57BL/6 mice infected with 10^6^ CFU of B1917GR 14 d.p.c. Cells were treated as described above. CD45iv^−^ CD45^+^ CD44^+^ CD69^+^ CD4^+^ T cells were sorted using BD FACS Aria II sorter (BD Biosciences). Purity of the isolated cells was checked prior to transfer (Supplementary Fig. 6). A total of 10^4^ cells were transferred intravenously to IL-17 KO mice 10 h.p.c. and 7 d.p.c.

### Statistical analyses

Statistical analyses were performed by non-parametric *T* tests, Kruskal–Wallis, or one-way analysis of variance tests and Mann–Whitney tests using the GraphPad Prism software. *P* values < 0.05 were considered significant.

### Reporting summary

Further information on research design is available in the Nature Research Reporting Summary linked to this article.

## Supplementary information

Supplementary Figures

Reporting Summary

## Data Availability

The authors confirm that the data supporting the findings of this study are available within the article and its Supplementary Materials. All other relevant data are available from the corresponding author on reasonable request.
