# Supplementary material for: Suppression of mucosal Th17 memory responses by acellular pertussis vaccines enhances nasal Bordetella pertussis carriage
Source: NPJ Vaccines. 2021 Jan 8;6:6. doi: 10.1038/s41541-020-00270-8 (PMC7794405; doi:10.1038/s41541-020-00270-8)
Supplement: Supplementary file 1 — Supplementary Figures [file 41541_2020_270_MOESM1_ESM.pdf]

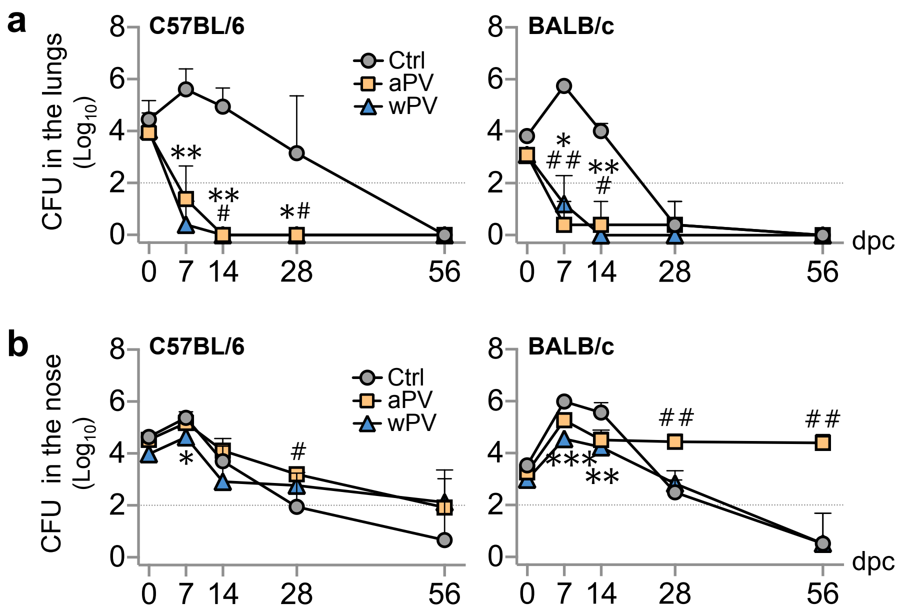

Supplementary Fig. 1: Nasal and lung colonization of mice challenged with a moderate dose of B1917GR. Six-week old C57BL/6 (left panels) and BALB/c mice (right panels) were immunized twice with 1/10 human dose of Infanrix (aPV, yellow squares) or Shan5 (wPV, blue triangles) vaccine at a four-week interval or left unvaccinated (Ctrl, grey dots) and nasally challenged with 10^5 CFU of B1917GR four weeks after the second immunization. Bacterial burden in the lungs (**a**) and noses (**b**) was determined by CFU counting at the indicated time points. Results shown are geometric means +SD. n = 3-4. Kruskal-Wallis tests were performed to compare aPV ($^{\#}$) and wPV (*) immunized mice to control mice. $*/^{\#}p < .05$, $**/^{\#\#}p < .01$, $***p < .001$. Only significant differences are indicated. Dashed lines correspond to the detection limit.

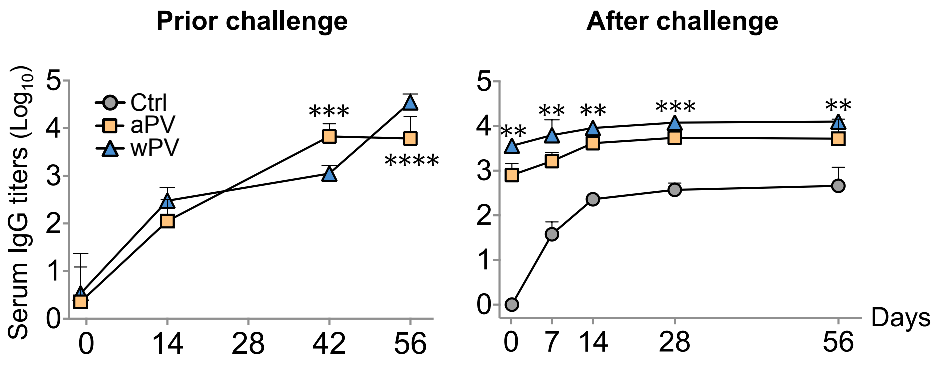

Supplementary Fig. 2: Serum IgG responses to whole *B. pertussis* extracts in aPV- and wPV-immunized mice before and after *B. pertussis* challenge. a BALB/c mice were vaccinated with aPV (in orange) or wPV (in blue) on days 0 and 28, and anti-*B. pertussis* IgG titers were determined at indicated times after vaccination. **b** At day 56 the mice were challenged with 10^6 CFU of B1917GR, and anti-*B. pertussis* IgG titers were determined at indicated times after challenge (right panel). Non-vaccinated challenged mice (in grey) served as controls. The results are expressed in Log_{10} titers, defined as the reciprocal of the dilution giving an optical density at 450 nm three times that of the blank. Results shown are geometric means +SD. n = 24-25 prior challenge. n = 4-5 after challenge. Kruskal-Wallis tests were performed to compare aPV- and wPV-immunized mice prior challenge and aPV and wPV (*) immunized mice to control mice after challenge. **$p < .01$; ***$p < 0.001$; ****$p < 0.0001$.

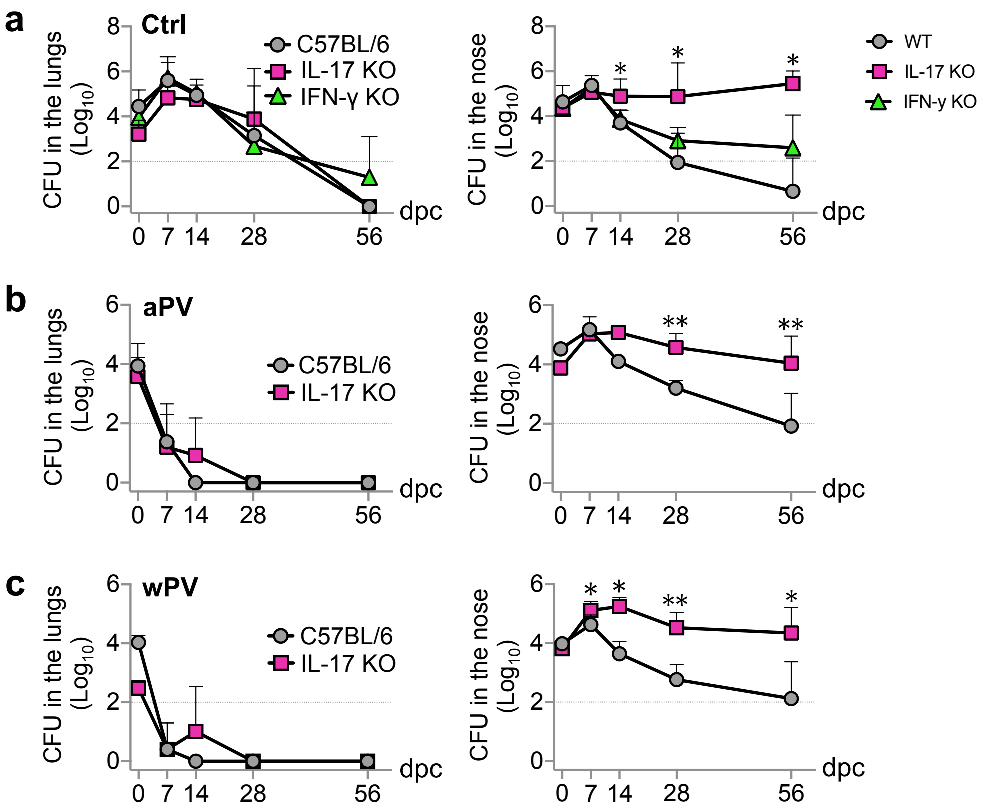

Supplementary Fig. 3: Lung and nasal colonization of IL-17 KO and IFN-γ KO mice challenged with a moderate dose of B1917GR. a C57BL/6 (in grey), IL-17 KO (in purple) and IFN-γ KO (in green) mice were infected with 10^5 CFU of B1917GR, and CFU numbers were counted in lungs (left panel) and noses (right panel) at indicated time points. **b** C57BL/6 (in grey), IL-17 KO (in purple) mice were immunized with aPV and then infected with 10^5 CFU of B1917GR, and CFU numbers were counted in lungs (left panel) and noses (right panel) at indicated time points. **c** C57BL/6 (in grey), IL-17 KO (in purple) mice were immunized with wPV and then infected with 10^5 CFU of B1917GR, and CFU numbers were counted in lungs (left panel) and noses (right panel) at indicated time points. Results shown are geometric means +SD. n = 3-5. Mann Whitney tests were performed to compare C57BL/6 mice and IL-17 (*) or IFN-γ (#) knockout mice. *p < .05, **p < .01. Only significant differences are indicated. Dashed lines correspond to the detection limit.

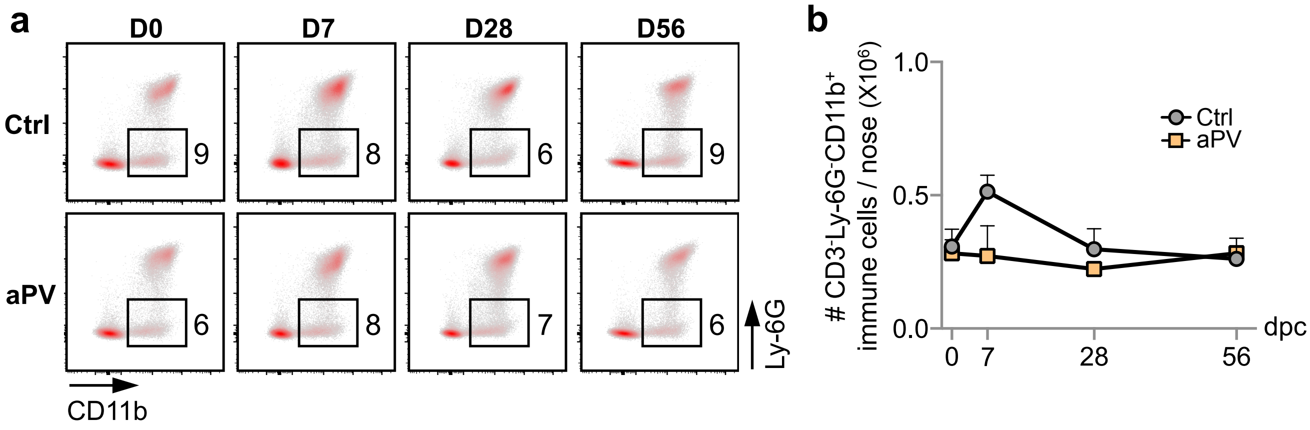

Supplementary Fig. 4: Immunization with aPV do not significantly impact the recruitment of CD3$^-$Ly-6G$^-$CD11b$^+$ immune cells in the nose of BALB/c mice. a Representative graphs showing the recruitment of CD3$^-$Ly-6G$^-$CD11b$^+$ immune cells (i.e. dendritic cells, monocytes and natural killer cells) in the nose of BALB/c mice vaccinated with aPV (lower panels) or left unvaccinated (upper panels) at different times after infection with 10^6 CFU of B1917GR. Numbers indicate percentages of events in each square. **b** Absolute numbers CD3$^-$Ly-6G$^-$CD11b$^+$ immune cells in the noses of BALB/c mice immunized with aPV (in yellow) or left un-immunized (in grey) at indicated time points after challenge with 10^6 CFU of B1917GR. Results shown are geometric means +SD. *n* = 3-5.

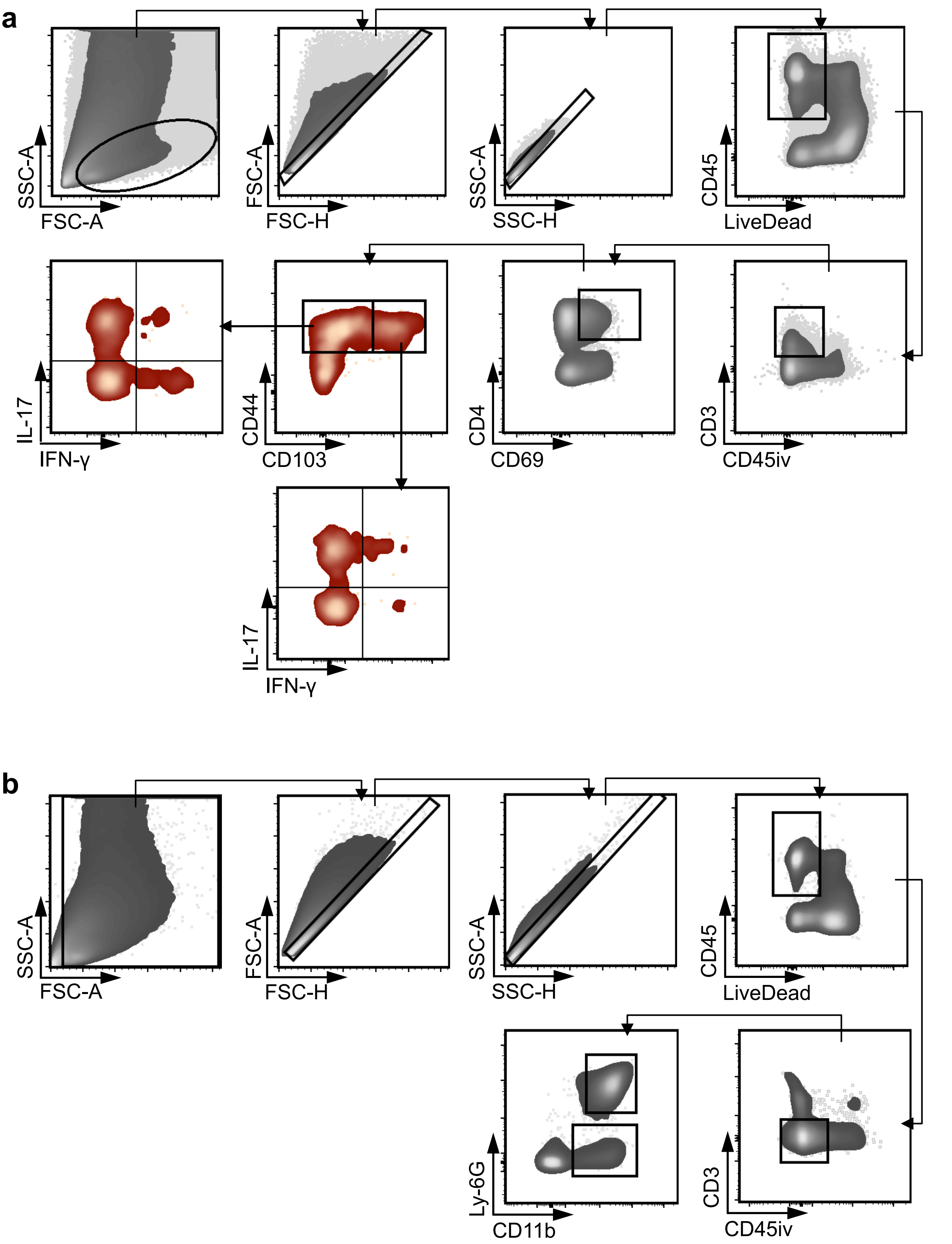

Supplementary Fig. 5: Gating strategies. Ten minutes before euthanasia, mice are injected intravenously with anti-CD45-PE (CD45iv) antibody enabling to discriminate circulating from infiltrated and resident immune cells. **a** Identification of $CD4^+$ T_{RM} cells (see **Fig. 2** and **Fig. 3**). $CD4^+$ T_{RM} cells express CD44, CD69 and/or CD103 (red density plots). Intracellular staining is performed to identify IL-17a$^+$ and IFN-γ$^+$ CD4 T_{RM} cells. **b** Identification of infiltrated neutrophils (CD11b$^+$, Ly-6G$^+$) and $CD3^-$Ly-6G$^-$CD11b$^+$ immune cells (see **Fig. 5** and **Supplementary Fig. 4**).

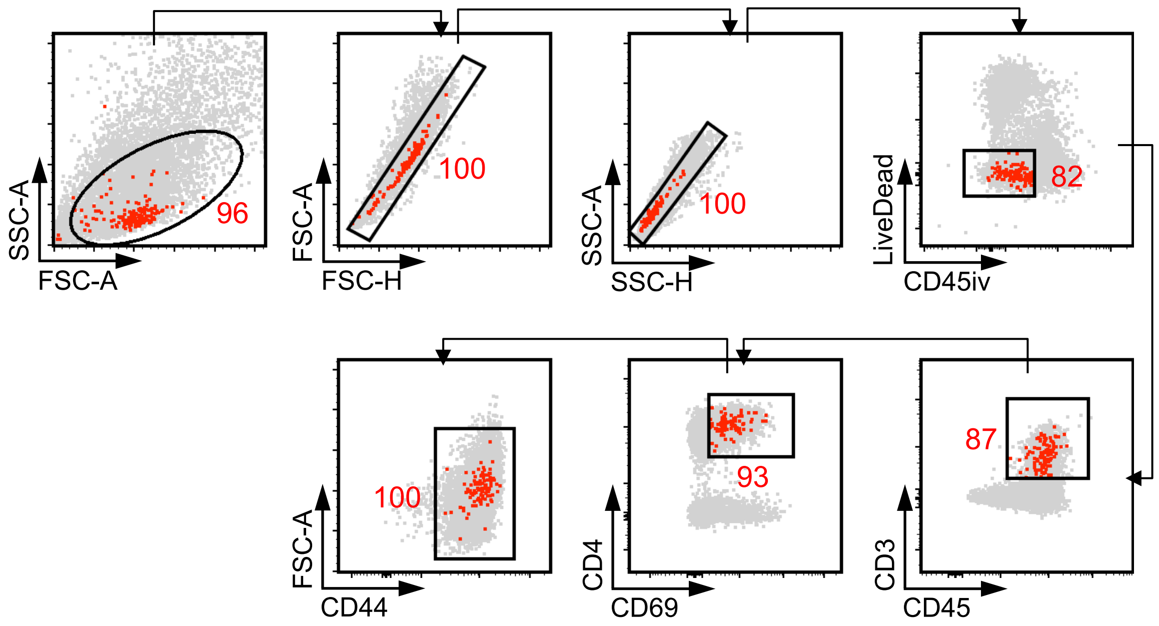

Supplementary Fig. 6: Isolation of Bp-induced CD4$^+$ T$_{RM}$ cells by cell sorting (see **Fig. 5**). CD4$^+$ T$_{RM}$ cells were purified from noses of C57BL/6 mice infected with 10^6 CFU of B1917GR 14 dpc. Grey dots represent total cells before sorting for CD45iv$^-$CD45$^+$CD44$^+$CD69$^+$ T cells. The red dots represent the purified cells, based on 100 cells acquired. Numbers in red represent percentages of purified CD45iv$^-$CD45$^+$CD44$^+$CD69$^+$ T cells in each gate.
