# Supplementary material for: Suppression of mucosal Th17 memory responses by acellular pertussis vaccines enhances nasal Bordetella pertussis carriage
Source: NPJ Vaccines. 2021 Jan 8;6:6. doi: 10.1038/s41541-020-00270-8 (PMC7794405; doi:10.1038/s41541-020-00270-8)
Supplement: Supplementary file 2 — Reporting Summary [file 41541_2020_270_MOESM2_ESM.pdf]

nature research

Corresponding author(s): Violaine Dubois

Last updated by author(s): Nov 25, 2020

Reporting Summary

Nature Research wishes to improve the reproducibility of the work that we publish. This form provides structure for consistency and transparency in reporting. For further information on Nature Research policies, see our Editorial Policies and the Editorial Policy Checklist.

Statistics

For all statistical analyses, confirm that the following items are present in the figure legend, table legend, main text, or Methods section.

n/a	Confirmed	
☐	☒	The exact sample size (n) for each experimental group/condition, given as a discrete number and unit of measurement
☐	☒	A statement on whether measurements were taken from distinct samples or whether the same sample was measured repeatedly
☐	☒	The statistical test(s) used AND whether they are one- or two-sided<br>*Only common tests should be described solely by name; describe more complex techniques in the Methods section.*
☒	☐	A description of all covariates tested
☒	☐	A description of any assumptions or corrections, such as tests of normality and adjustment for multiple comparisons
☐	☒	A full description of the statistical parameters including central tendency (e.g. means) or other basic estimates (e.g. regression coefficient) AND variation (e.g. standard deviation) or associated estimates of uncertainty (e.g. confidence intervals)
☐	☒	For null hypothesis testing, the test statistic (e.g. F, t, r) with confidence intervals, effect sizes, degrees of freedom and P value noted<br>*Give P values as exact values whenever suitable.*
☒	☐	For Bayesian analysis, information on the choice of priors and Markov chain Monte Carlo settings
☒	☐	For hierarchical and complex designs, identification of the appropriate level for tests and full reporting of outcomes
☒	☐	Estimates of effect sizes (e.g. Cohen's d, Pearson's r), indicating how they were calculated

Our web collection on statistics for biologists contains articles on many of the points above.

Software and code

Policy information about availability of computer code

Data collection	Flow cytometry data were collected with a LSR Fortessa using the BD Diva Software (BD Biosciences).
Data analysis	Flow cytometry data were analyzed with the Flowjo Software (v10, TreeStar). Statistical analyses were performed with Graphpad Prism 8 software.

For manuscripts utilizing custom algorithms or software that are central to the research but not yet described in published literature, software must be made available to editors and reviewers. We strongly encourage code deposition in a community repository (e.g. GitHub). See the Nature Research guidelines for submitting code & software for further information.

Data

Policy information about availability of data

All manuscripts must include a data availability statement. This statement should provide the following information, where applicable:

- Accession codes, unique identifiers, or web links for publicly available datasets
- A list of figures that have associated raw data
- A description of any restrictions on data availability

The authors confirm that the data supporting the findings of this study are available within the article and its supplementary materials.

Field-specific reporting

Please select the one below that is the best fit for your research. If you are not sure, read the appropriate sections before making your selection.

☒ Life sciences ☐ Behavioural & social sciences ☐ Ecological, evolutionary & environmental sciences

For a reference copy of the document with all sections, see nature.com/documents/nr-reporting-summary-flat.pdf

Life sciences study design

All studies must disclose on these points even when the disclosure is negative.

Sample size	A minimum of 5 mice per group was found to provide reproducible results (and relevant by statistical analyses).
Data exclusions	No data were excluded.
Replication	All attempts at replication were successfull.
Randomization	All animals in this study were six-week old or more. For some experiments, we mixed males and females and we checkedB that there was no sexe effect on the concerning experiments.
Blinding	Blinding was not relevant for this study since the experimenters could not affect the outcomes.

Reporting for specific materials, systems and methods

We require information from authors about some types of materials, experimental systems and methods used in many studies. Here, indicate whether each material, system or method listed is relevant to your study. If you are not sure if a list item applies to your research, read the appropriate section before selecting a response.

Materials & experimental systems

n/a	Involved in the study	
☐	☒	Antibodies
☒	☐	Eukaryotic cell lines
☒	☐	Palaeontology and archaeology
☐	☒	Animals and other organisms
☒	☐	Human research participants
☒	☐	Clinical data
☒	☐	Dual use research of concern

Methods

n/a	Involved in the study	
☒	☐	ChIP-seq
☐	☒	Flow cytometry
☒	☐	MRI-based neuroimaging

Antibodies

Antibodies used	- CD45-PE antibody (ebiosciences) (SEE REF 12-9459-42, ThermoFisher)<br>- CD45-APC (eBioscience) (SEE REF 17-0452-82, ThermoFisher)<br>- CD44-BV605 (eBiosciences) (SEE REF 103047, BioLegend)<br>- CD4-BV785 (BioLegend) (SEE REF 100552, BioLegend)<br>- CD103-PE-CF594 (BD Biosciences) (SEE REF 565849, BD Biosciences)<br>- IL-17-V450 (BD Biosciences) (SEE REF 560522)<br>- IFN-γ-PE-Cy7 (eBiosciences) (SEE REF 25-7319-41, ThermoFisher)<br>- Ly-6G-PE-CF594 (BD Biosciences) (SEE REF 562700, BD Biosciences)<br>- CD11b-BV605 (BD Biosciences) (SEE REF 563015, BD Biosciences)
Validation	For validation of the antibodies please refer to the refrences provided above and to the gating strategy displayed in Supplementary Figure 4. All the FACS experiments were validated by our collaborator Dr. Helene Bauderlique-Roy, flow cytometry engineer.

Animals and other organisms

Policy information about studies involving animals; ARRIVE guidelines recommended for reporting animal research

Laboratory animals	BALB/cJ and C57BL/6J mice were used. As well as IL-17 knockout and IFN-γ knockout mice were generated in the C57BL/6 background. All mice were six-week old or more and some experiments males and females were used in order to avoid sacrifice of mice bread in our facilities.
Wild animals	The study did not involve wild animals.

Field-collected samples	The study did not imply fielf-collected samples.
Ethics oversight	All the animal experiments were carried out in accordance with the guidelines of the French Ministry of Research, and the protocols were approved by the Ethical Commitees of the Region Nord Pas de Calais and the Ministry of Research (Agreement number APAFIS #2019052015506229 V4).

Note that full information on the approval of the study protocol must also be provided in the manuscript.

Flow Cytometry

Plots

Confirm that:

☒ The axis labels state the marker and fluorochrome used (e.g. CD4-FITC).

☒ The axis scales are clearly visible. Include numbers along axes only for bottom left plot of group (a 'group' is an analysis of identical markers).

☒ All plots are contour plots with outliers or pseudocolor plots.

☒ A numerical value for number of cells or percentage (with statistics) is provided.

Methodology

Sample preparation	Ten minutes before euthanasia, mice were injected intravenously with 10 µg of anti-CD45-PE antibody (ebiosciences) to allow for the distinction of circulating T cells (CD45-PE positive) and resident T cells or infiltrated immune cells (CD45-PE negative). Nasal tissue was treated as described above. Red blood cells were lysed using ACK lysing buffer (Gibco). Isolated cells were stimulated with 50 ng/ml PMA (InVivoGen) and 500 ng/ml ionomycin (Sigma) in the presence of 5 µg/ml brefeldin A (Sigma) for 4 h at 37 °C. Cells were incubated with dead cell stain kit LIVE/DEAD Aqua (Invitrogen) first as recommended by the supplier, then with Fc block (BD biosciences) (1:50), followed by surface staining with the following fluorochrome-conjugated antibodies: CD45-APC, CD69-FITC, CD8-AF700, CD3-APC-ef780 (eBioscience), CD44-BV605, CD4-BV785 (BioLegend), and CD103-PE-CF594 (BD Biosciences). For the detection of intracellular cytokines, cells were fixed and permeabilized using a FoxP3 Transcription Factor staining buffer set (eBioscience), and stained with the following antibodies: IL-17-V450 (BD Biosciences) and IFN-γ-PE-Cy7 (eBioscience). Fluorescence minus one samples were used as controls.
Instrument	Fluorescence-activated cell sorting samples were acquired on a LSR Fortessa using the BD Diva Software (BD Biosciences).
Software	Samples were analyzed using the Flowjo Software (v10, TreeStar).
Cell population abundance	Purity of cell population of interest is illustrated in Supplementary Figure 5.
Gating strategy	Lymphocytes cells are gated, that are SSC low cells. Only the singlets are considered. We identify CD3+ cells in alive CD45iv negative cells (ie resident T cells). CD4+CD69+ Tcells are screened for the expression of CD103 and CD44. Activation of CD103+CD44+ and CD103-CD44+ cells is measured by in vitro stimulation and intracellular stainings of IL-17A and IFN-γ.

☒ Tick this box to confirm that a figure exemplifying the gating strategy is provided in the Supplementary Information.
